# Illusory Reversal of Causality between Touch and Vision has No Effect on Prism Adaptation Rate

**DOI:** 10.3389/fpsyg.2012.00545

**Published:** 2012-12-11

**Authors:** Hirokazu Tanaka, Kazuhiro Homma, Hiroshi Imamizu

**Affiliations:** ^1^School of Information Science, Japan Advanced Institute of Science and TechnologyNomi, Ishikawa, Japan; ^2^Nagaoka University of TechnologyNiigata, Japan; ^3^Center for Information and Neural Networks, National Institute of Information and Communications TechnologyOsaka, Japan; ^4^Cognitive Mechanisms Laboratories, Advanced Telecommunication Research Institute InternationalKyoto, Japan

**Keywords:** motor adaptation, subjective simultaneity, physical simultaneity, temporal adaptation, spatial adaptation, illusory reversal

## Abstract

Learning, according to Oxford Dictionary, is “to gain knowledge or skill by studying, from experience, from being taught, etc.” In order to learn from experience, the central nervous system has to decide what action leads to what consequence, and temporal perception plays a critical role in determining the causality between actions and consequences. In motor adaptation, causality between action and consequence is implicitly assumed so that a subject adapts to a new environment based on the consequence caused by her action. Adaptation to visual displacement induced by prisms is a prime example; the visual error signal associated with the motor output contributes to the recovery of accurate reaching, and a delayed feedback of visual error can decrease the adaptation rate. Subjective feeling of temporal order of action and consequence, however, can be modified or even reversed when her sense of simultaneity is manipulated with an artificially delayed feedback. Our previous study (Tanaka et al., 2011; Exp. Brain Res.) demonstrated that the rate of prism adaptation was unaffected when the subjective delay of visual feedback was shortened. This study asked whether subjects could adapt to prism adaptation and whether the rate of prism adaptation was affected when the subjective temporal order was illusory reversed. Adapting to additional 100 ms delay and its sudden removal caused a positive shift of point of simultaneity in a temporal order judgment experiment, indicating an illusory reversal of action and consequence. We found that, even in this case, the subjects were able to adapt to prism displacement with the learning rate that was statistically indistinguishable to that without temporal adaptation. This result provides further evidence to the dissociation between conscious temporal perception and motor adaptation.

## Introduction

Learning from experience implicitly assumes that the temporal order of an action and a consequence is causally preserved. It is thus natural to expect that action-consequence timing plays an important role in learning and that a delay in consequence feedback would affect the course of motor learning. Standard theories for learning sequential tasks assume temporal proximity as a critical factor in determining a causal relationship between actions and consequences (Sutton and Barto, 1998). In reinforcement learning, for instance, an action and a reward are associated with a so-called eligibility trace that is elicited at the moment of action and is decreased gradually. Another example of action-consequence timing can be found in motor adaptation to a visual displacement induced by wedge prisms that bend the light path. Initial movements miss the target by an amount proportional to the prisms diopter, but accurate reaching is gradually recovered with successive trials (Martin et al., 1996). The timing between arm movements and the feedback of the visual errors plays an essential role in the rate of prism adaptation. Previous psychophysics studies demonstrated that a short delay of endpoint error feedback in prism adaptation decreases the learning rate both in humans (Kitazawa et al., 1995) and monkeys (Kitazawa and Yin, 2002). Most remarkable was that an almost imperceptibly short delay of 50 ms decreased the learning rate significantly. These results indicate that the motor system employs an accurate time keeping system between an action and an outcome during motor control and motor adaptation.

Recent lines of psychophysical studies, on the other hand, have documented that our subjective perception of temporal duration and temporal order is not innately fixed but rather flexibly modifiable after passively exposed to stimuli that are temporally out of sync with a persistent lag (for review, see Vroomen and Keetels, 2010). After adapting to asynchronized stimuli, the psychometric curve is shifted so that the point of subjective simultaneity (PSS) moves toward the exposed lag (lag adaptation; Fujisaki et al., 2004) or away from the exposed lag (Bayesian-like adaptation; Miyazaki et al., 2006). A recent study demonstrated that, in audiovisual temporal order judgments (TOJ), Bayesian calibration is at work behind lag adaptation (Yamamoto et al., 2012), so these adaptation mechanisms are not mutually exclusive but rather competitive to each other. This effect of temporal adaptation has been reported within a single modality and across multiple modalities, indicating that our sense of timing is not innately determined but instead flexibly adaptive.

Temporal adaptation is observed not only in passive exposure of cross-modal stimulus pairs but also between voluntary actions and sensory consequences (Cunningham et al., 2001; Stetson et al., 2006; Heron et al., 2009; Wenke and Haggard, 2009; Keetels and Vroomen, 2012), often referred to as intentional binding (Haggard et al., 2002). Of particular interest is an intriguing perception after adapting to a delay between an action and a consequence. After adapting to a visual feedback delay in response to a button press, a sudden removal of the delay causes a sensation that the visual feedback occurred in prior to the button press, a phenomenon known as illusory reversal (Cunningham et al., 2001; Stetson et al., 2006; Heron et al., 2009). This reversal can be quantitatively evaluated by comparing psychometric curves in a TOJ experiment. With a delay of 100 ms, the PSS reportedly shifted by 40 ms (Stetson et al., 2006).

An essential question in studying neural temporal processing is whether there is a single “master clock” that controls timings regardless of stimulus characteristics (i.e., visual, auditory, or tactile) or there are “distributed clocks” that control timings depending stimulus modalities and/or behavioral contexts (see Buonomano and Karmarkar, 2002 for review). There is experimental evidence for the master clock hypothesis. Individual differences in produced intervals for finger and foot movements correlated with the acuity of auditory timing judgment (Keele et al., 1985), indicating common timing systems among perceptual and motor systems. The distributed-clock hypothesis, on the other hand, is supported by the finding that the performance of sub-second interval discrimination tasks was significantly worse when intervals were bounded by intermodal stimuli (i.e., a tone and a flash of light) than when intervals were bounded by unimodal stimuli (i.e., two tones or two flashes of light; Rousseau et al., 1983). Recently, degrees of temporal adaptation were evaluated with various pairs of multimodal stimuli (auditory/visual, auditory/tactile, and visual/tactile), and adaptation to auditory-visual stimuli was larger than that to auditory-tactile or visual-tactile pairs (Harrar and Harris, 2008). Both hypotheses thus have been supported by experimental findings, but in what conditions and contexts the brain uses a single clock or multiple clocks remains unanswered.

Although temporal perception has long been studied in both perceptual and motor processing, it is little investigated how temporal perception affects the courses of motor adaptation (Tanaka et al., 2011; Honda et al., 2012). When temporally associating an action and a consequence in motor learning, there are two possible timings: subjective and physical timings. Our previous study asked whether the learning rate in prism adaptation depends on subjective delay or physical delay (PD; Tanaka et al., 2011). Psychometric curves in a TOJ experiment shifted by about 40 ms after adapting to a persistent delay of 100 ms of visual feedback stimulus. The learning rate in prism adaptation was not influenced by prior lag adaptation but was predicted by PD (i.e., delay during a prism adaptation session). Therefore, in that study, subjective shortening of feedback delay had no effect on the learning rate in prism adaptation. The current study extends our previous study by examining the learning rate in prism adaptation when subjective temporal order between an action and a consequence is illusory reversed. Subjects adapted to a temporal delay (100 ms) of visual feedback in finger pointing movement, and then adapted to a visual displacement with the delay removed. This caused an illusory reversal of touching and visual feedback. By evaluating the learning rate of prism adaptation, the effect of illusory reversal on motor adaptation was studied.

## Materials and Methods

### Subjects

Six right-handed male adults from our institution and a nearby university (age: 21–46) participated in the experiments. This study was approved by an institutional ethics committee, and a written consent form was obtained from all subjects. They reported no known neurological history and had normal or corrected-to-normal vision.

### Experimental setting

**Figure 1:**
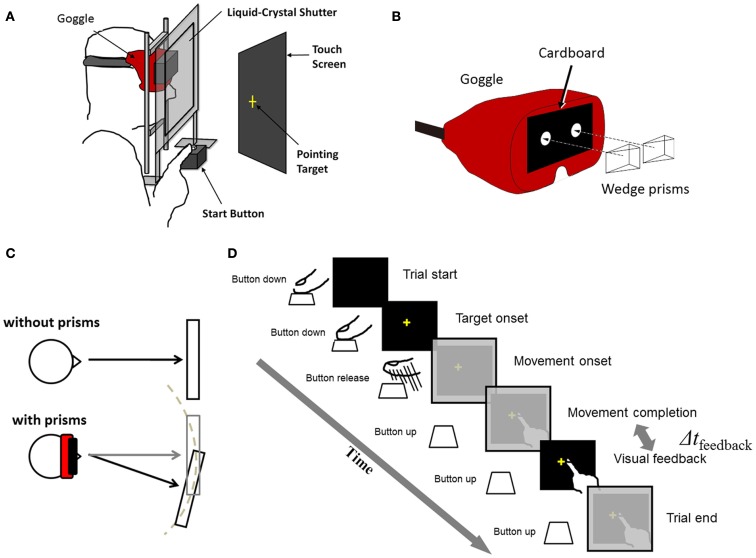
**(A)** Schematic of experimental setting. **(B)** Goggles restricting visual field of subjects. Wedge prisms were put on the goggles in adaptation conditions. **(C)** Display locations (gray rectangles) with and without prism, adjusted so that the target location and the visual feedback fall within the central visual field. **(D)** Time flow of a single trial. Δ*t*_feedback_ is the interval from movement completion to visual feedback and is adjusted in various conditions.

Detailed descriptions about the experimental setup were found in our previous study (Tanaka et al., 2011), so a brief summary is provided here. The experimental setup consisted of a 17″ CRT (85 Hz refresh rate: CV772X, TOTOKU Electric Coop, Tokyo, Japan) for presenting visual stimuli, a touch screen on the display that measured pointed locations (resolution was 3 mm × 3 mm), a custom-made liquid-crystal shutter for controlling the timing of visual feedback (Takei Coop, Niigata, Japan), a button for measuring movement onset time, and a mouse for recording subjects’ responses (Figure 1A). The subjects sat comfortably in front of the desk with their heads stabilized on a chin rest. The distance between the subject’s face and the CRT screen was 30 cm. Subjects wore goggles whose glasses were removed and front was covered with a black cardboard having two holes (each diameter = 1.7 cm; Figure 1B). The holes restricted subjects’ vision in the central field (visual angle = 27.3°). The goggles were worn for all trials including adaptation and test trials for Experiment 1 and baseline, adaptation, washout trials for Experiment 2. We restricted the vision to the central field so that the subjects could not see the edges of the CRT screen; otherwise, the subjects could have noticed the prism displacement by observing the screen edges. In the adaptation trials, wedge prisms were put on the goggles covering the holes of the cardboard (Figure 1B) and inducing rightward visual displacement (15 diopter or 10.7 cm on the screen). The CRT position was adjusted with or without the prisms so that targets appeared within the central visual field (Figure 1C). Due to the holes and the position adjustment, the size of the visual field was constant and independent of existence the prisms. A target was displayed at a random location (taken from a uniform distribution) in a 4 cm × 4 cm at the center of display. The starting button was placed 20 cm in front of the screen. The distance between the button and the center of the display was 30 cm. For visual stimulus-presentation, recoding of behavioral responses and control of the shutter, in-house Matlab codes (MathWorks, MA, USA) based on the Psychtoolbox extension (Brainard, 1997; Pelli, 1997). By using a commercial high-speed digital camera (EX-F1, Casio, Japan, maximum 1,200 frames per second), we measured a temporal delay from the moment the subject’s finger contacted with the touch screen to the moment the visual feedback was made visible to the subject. When no delay included in the stimulus-presentation code, the measured delay was 36.4 (SD 5.08) ms. This inevitable delay was referred to as the minimum delay (MD). When a 100-ms delay was imposed, the measured delay was 136.4 (SD 4.67) ms.

### Psychophysical experiments

Commonly in all conditions described below, the subjects were instructed, in a single trial, to make a rapid pointing movement with their right arm as soon as a visual target (a yellow cross, 1 cm × 1 cm) was presented on the screen (Figure 1D). The vision of the hand was provided not during the movement but after the movement. Several conditions were tested by changing the timing of visual feedback (Δ*t*_feedback_ in Figure 1D) and/or by imposing the visual displacement with the prisms. The visual feedback was provided immediately (36 ms) or with an additional 100 ms delay (thus 136 ms) after the subjects touched the screen. The visual displacement was imposed when subjective temporal order was tested in the delay adaptation experiment (Experiment 1) and when prism adaptation was measured in the prism adaptation experiment (Experiment 2). Otherwise, the veridical vision (i.e., no visual displacement) was imposed. Before participating in the experiments, the subjects went through training sessions until they achieved accuracy (50% of trials with movement error ≤1 cm) and timing criteria [within both the response-time (200 ms) and movement-time (250 ms) limits in no less than 90% of trials in a single training session].

#### Experiment 1: delay adaptation experiment

The first experiment tested whether TOJ adapted in a large-amplitude movement involving full upper-limb movement under conditions of laterally displaced vision. A single session consisted of 60 initial delay adaptation trials (without prism) followed by 20 subsequent test trials (with prism). During the delay adaptation trials the feedback delay was 36 ms for the MD condition and was 136 ms for the delay adaptation condition. In the test trials, the delay was the average movement duration during the initial adaptation trials (${\bar{t}}_{\text{movement}}$) plus a Gaussian random number (mean 0 ms, SD 80 ms), so the visual feedback appeared either before or after the actual touch on the screen (feedback timings summarized in Table 1). Subsequently, during the test trials the subjects reported, with a computer mouse held in their left hands, whether the visual feedback was felt before or after the moment they touched the screen.

**Table 1:** **Feedback delays (Δ*t*_feedback_) used for the minimum delay and delay adaptation conditions**.

Condition	Adaptation (60 trials; ms)	Test (20 trials)
Minimum delay	36	${\bar{t}}_{\text{movement}}+N\left(0\text{ ms, }80\text{ ms}\right)$
Delay adaptation	136	${\bar{t}}_{\text{movement}}+N\left(0\text{ ms, }80\text{ ms}\right)$

*During the test trials (gray shaded column) a visual shift was imposed whereas no visual shift was imposed during the adaptation trials*.

#### Experiment 2: prism adaptation

The second experiment investigated the effect of temporal adaptation on the adaptation rate in prism adaptation. One session consisted of (1) 60 baseline trials, (2) 30 adaptation trials, and (3) 30 washout trials, a total of 120 trials. Only during the adaptation trials, the visual displacement was imposed by the prisms.

There were four conditions of variable feedback timings as summarized in Table 2. Each subject was tested two sessions for each condition, totally participating in eight sessions, and the order of the four conditions was randomized subject by subject. In the MD condition the delay was minimal (36 ms) through baseline, prism adaptation, washout trials. Therefore, learning coefficients obtained in this condition were regarded as a reference compared to those in other conditions. In the PD condition an additional delay (136 ms) was added to the timings of visual feedback during the prism adaptation trials so that the effect of delayed visual feedback on prism adaptation could be evaluated. The delay adapted (DA) and subjectively reversed (SR) conditions were specifically designed to evaluate the effect of lag adaptation on the learning rate during prism adaptation. In the DA condition, the 136-ms delay was imposed during both the baseline and prism adaptation trials, so the effect of temporal adaptation was evaluated by comparing with the PD condition. In the SR condition, the 136-ms delay was imposed to induce temporal adaptation during the baseline trials and was then reduced to the MD during the prism adaptation trials; in this condition, subjective illusory reversal between screen touch and visual feedback was expected. All the subjects completed two sessions of each of the four conditions (i.e., totally eight sessions).

**Table 2:** **Actual feedback delays used for the minimum delay, physical delay, delay adapted, and subjective delay conditions**.

Condition	Baseline (60 trials; ms)	Adaptation (30 trials; ms)	Washout (30 trials; ms)
Minimum delay (MD)	36	36	36
Physical delay (PD)	36	136	36
Delay adapted (DA)	136	136	36
Subjectively reversed (SR)	136	36	36

*During the adaptation trials (gray shaded column) a visual shift was imposed whereas no visual shift was imposed during the baseline or washout trials*.

### Data analysis

All the analyses described below were performed Matlab (MathWorks, MA, USA). For the delay adaptation experiment (Experiment 1), the probability that the visual feedback was judged to be after the screen touch was computed from the subjects’ responses (Stetson et al., 2006; Tanaka et al., 2011). We then fitted a sigmoid function

$$\begin{array}{rcll} & \mathrm{Pr}\text{ob}\left[\text{visual feedback judged after touch | }t\right] & & \\ & \;=\frac{1}{1+\exp\left(\frac{t-t_{0}}{b}\right)}, & & \text{(1)} \end{array}$$

where *t* denotes the time of visual feedback measured from the time of touching the screen. Namely, a positive value of *t* indicates that the visual feedback appears after a screen touch, and a negative value of *t* indicates otherwise. *t*_0_ and *b* are the intercept and the slope, respectively. In particular, *t*_0_ is the time when the probability becomes 0.5, thereby the PSS. Therefore, by comparing the values of *t*_0_ in the baseline condition and in the delay adaptation condition, the shift of PSS was evaluated.

For the prism adaptation experiment (Experiment 2), the learning rate during adaptation trials was estimated by a discrete-model approach (Kitazawa et al., 1995; Yin and Kitazawa, 2001; Tanaka et al., 2011), in which a learning coefficient (*k*) is defined as a portion of an error in previous trial that reduces an error in current trial:

(2) $$\varepsilon_{n}=\varepsilon_{n-1}-k\varepsilon_{n-1}$$

Here, ε*_n_* denotes a horizontal error in *n*-th trial. The coefficient, by definition, takes a value between 0 and 1. Rewriting Eq. 2 into

(3) $$\varepsilon_{n}-\varepsilon_{1}=-k\overset{n-1}{\underset{i-1}{\sum }}\varepsilon_{i}$$

suggests that the coefficient is a slope of a line connecting the origin (0, 0) and a point $\left(\overset{n-1}{\underset{i=1}{\Sigma }}\varepsilon_{i},\varepsilon_{n}-\varepsilon_{1}\right)$, so the learning coefficient for one session can be obtained by minimizing a least-squares sum

(4) $$\overset{30}{\underset{n=2}{\sum }}{\left(\varepsilon_{n}-\varepsilon_{1}+k\overset{n-1}{\underset{i=1}{\sum }}\varepsilon_{i}\right)}^{2}.$$

For each condition the subjects attended two sessions, so learning coefficients for the two sessions were averaged to represent the learning coefficient of one subject for one condition. The coefficients were computed for the four conditions (denoted as *k*_MD_, *k*_PD_, *k*_DA_, and *k*_SR_, respectively).

## Results

### PSS shift found in delay adaptation experiment

Experiment 1 (delay adaptation experiment) checked whether and what amount the PSS was shifted in full arm movements under visual displacement. Psychometric curves in the TOJ task were computed in both baseline and delay adaptation conditions. For all subjects, PSS shift was positively shifted toward the timing of visual feedback (lag adaptation) with mean 40.1 ms and SE 8.5 ms (see individual PSS data summarized in Table 3). These shifts were statistically significant [*t*(5) = 4.74, *p* = 0.005]. Therefore, the subjective simultaneity was shifted due to the delayed visual feedback.

**Table 3:** **Summary of results of experiment 1 (PSS shift) and experiment 2 (*k*_MD_, *k*_PD_, *k*_DA_, and *k*_SR_)**.

Subject number	PSS shift (ms)	*k*_MD_	*k*_PD_	*k*_DA_	*k*_SR_
1	6.3	25.0	15.9	15.4	18.1
2	29.7	7.3	3.4	3.2	7.5
3	42.9	4.1	2.8	2.5	6.7
4	44.0	7.2	5.1	4.9	6.3
5	49.9	6.7	6.4	4.3	6.5
6	67.9	23.9	19.8	11.15	33.3
Group (mean and SE)	40.1 (SE 8.5)	12.3 (SE 3.5)	8.9 (SE 2.9)	6.9 (SE 2.1)	13.1 (SE 4.5)

*The subjects are arranged in order of increasing PSS shifts*.

### No effect of illusory reversal on prism adaptation rate in prism adaptation experiment

**Figure 2:**
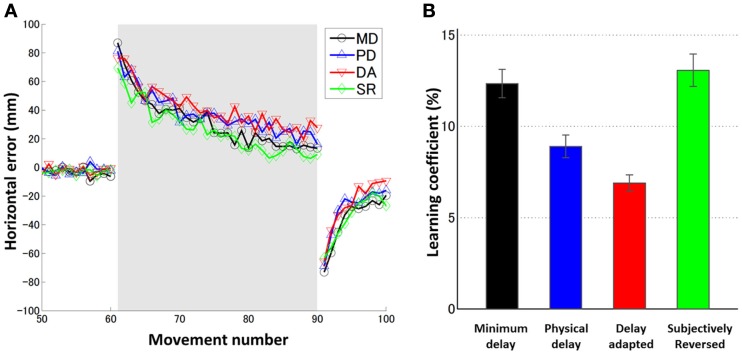
**(A)** Learning curves averaged over six subjects for four conditions (MD: black circle, PD: blue upward-pointing triangle, DA: red downward-pointing triangle, and SR: green diamond). The gray shaded trials (movements 61–90) indicate the block of prism adaptation. **(B)** Learning coefficients for six individual subjects for four conditions. Error bars indicate SE.

In Experiment 2 (prism adaptation experiment) we asked whether the rate of prism adaptation was affected by illusory reversal between screen touch and visual feedback. Four conditions (MD, PD, DA, and SR conditions) were tested. Figure 2 and Table 3 summarize learning curves averaged over all subjects and learning coefficients of individual subjects. The feedback delay during the adaptation trials (61–90 trials) was 36 ms in the MD and SR conditions, and 136 ms in the PD and DA conditions (see Materials and Methods and Table 2). We applied a one-way repeated measures ANOVA to learning coefficients (learning rates), and found a significant effect of condition [*F*(3, 15) = 3.89, *p* = 0.03]. Then we investigated which pair of conditions is significantly different in learning rates using a *t*-test in which *p*-values were corrected for number of comparisons (possible pairs of the four conditions = 6) using the Bonferroni method.

In order to assess the effect of delay adaptation in prior to prism adaptation, the learning coefficients in the MD and SR conditions and the learning coefficients in the PD and DA conditions should be statistically compared. The learning rates in the PD and DA conditions were not statistically significantly different [*t*(10) = 0.56, *p* > 0.5 corrected (0.59 × 6), N.S.], confirming the results of our previous study (Tanaka et al., 2011). Therefore, in these two conditions, there was no significant effect of delay adaptation on the learning rates. There was no statistically significant difference between the learning rates in the MD and SR conditions either [*t*(10) = 0.12, *p* > 0.5 corrected (0.90 × 6), N.S.], so again in these two conditions no significant effect of delay adaptation on the learning rates was observed. Taken together, delay adaptation, even when illusory reversal was expected, had no significant effects on the learning rates during prism adaptation.

We also investigated, when no temporal adaptation was induced, how the visual feedback delay during prism adaptation affected the adaptation time courses. The learning rate in the PD condition was smaller than that in the MD condition, for all six subjects. However, the above *t*-test could not detect a significant difference [*t*(10) = 1.24, *p* > 0.5 corrected (0.24 × 6), N.S.]. Because our previous study using the same experimental setting (Tanaka et al., 2011) as well as studies from the other group (Kitazawa et al., 1995; Kitazawa and Yin, 2002) have indicated that the additional delay of 100 ms during prism adaptation was sufficient to cause a significant decrease in the learning rate, we conducted an additional analysis using a paired *t*-test only for the comparison between the PD and MD conditions to confirm the effect of the delay. We found a statistically significant difference [*t*(5) = 2.71, *p* = 0.04], suggesting decrease in learning rate due to delay of the visual feedback.

## Discussion

We investigated whether and how temporal lag adaptation affected subsequent motor adaptation by quantifying the learning coefficients of prism adaptation under various timings of visual feedback. Specifically, by imposing a visual feedback delay of additional 100 ms during delay adaptation trials and then removing the delay during prism adaptation trials, an illusory reversal between an action (button press) and a consequence (visual feedback) was induced. Even with the subjective reversal, the learning coefficient was not found to be statistically different from that calculated in the baseline condition. This result was in line with our previous result that subjective shortening of visual feedback delay did not have any significant effect on the rate of prism adaptation (Tanaka et al., 2011). Taken together, we conclude that the subjective temporal adaptation has no significant effect on adaptation to visual displacement by wedge prisms, thereby adding supporting evidence for the distributed-clock hypothesis, i.e., independent timing systems for perception of subjective causality and adaptation to prism displacement.

Our finding of independence between temporal adaptation and prism adaptation appears to be remarkable for the following three reasons. First, there are previous studies reporting the influence of a perceptual or cognitive task on a simultaneous motor task. Dual task paradigms composed of a sensorimotor task and a cognitive task, for example, usually impair the performance of sensorimotor adaptation (Redding et al., 1985; Taylor and Thoroughman, 2007). In addition, reaction times of a motor task are significantly influenced by subjective judgment of temporal order (Cardoso-Leite et al., 2007). Second, brain areas that are responsible for temporal perception of sub-second overlap those that are responsible for motor control (Lewis and Miall, 2003a,b). In particular, the cerebellum is associated with various tasks that require precise timing that is equal to or shorter than a few seconds (Ivry, 1996; Ivry and Spencer, 2004). The cerebellum is, on the other hand, associated with sensorimotor adaptation such as prism adaptation (Martin et al., 1996). Finally, a range of temporal delays that is detrimental for prism adaptation (50–500 ms; Kitazawa et al., 1995; Kitazawa and Yin, 2002) is conspicuously similar to that for illusory reversal (100–200 ms; Cunningham et al., 2001; Stetson et al., 2006). From these lines of evidence one might expect that adapted temporal perception can affect the learning course of prism adaptation. The results reported here, however, showed no significant effect of illusory reversal on the learning coefficients of prism adaptation.

Neural correlates of synchronous detection and temporal binding among cross-modal sensory stimulus onsets have been well investigated especially for audiovisual stimulus pairs (Bushara et al., 2001, 2003; Dhamala et al., 2007; Noesselt et al., 2007; Powers III et al., 2012). Cortical and subcortical areas that are activated for sub-second temporal tasks include insular, parietal, prefrontal, cerebellar, and superior colliculus. Depending on subjective reports of synchrony and asynchrony between stimuli, distributed sub-networks among these areas were distinctly employed. In contrast, a relatively few studies have been reported for neural correlates related to temporal binding between voluntary actions and consequential feedback stimuli (Stetson et al., 2006; Stekelenburg et al., 2011). Stetson et al. (2006) suggested multiple timing systems and argued that the observed activation in the anterior cingulate cortex reflected subliminal error detection or error detection between subjective and physical intervals. Although there are some overlaps between areas involved in temporal binding of cross-modal sensory stimuli and areas involved in temporal binding of action and consequence, it is not understood whether the two kinds of temporal processing are processed by shared or distinct neural mechanisms. Further studies are necessary to clarify the neural mechanisms that underlie temporal binding of sensory signals and motor control.

## Conflict of Interest Statement

The authors declare that the research was conducted in the absence of any commercial or financial relationships that could be construed as a potential conflict of interest.
